# Psychophysiological and behavioral responses to descriptive labels in modern art museums

**DOI:** 10.1371/journal.pone.0284149

**Published:** 2023-05-03

**Authors:** Serena Castellotti, Ottavia D’Agostino, Angelica Mencarini, Martina Fabozzi, Raimondo Varano, Stefano Mastandrea, Irene Baldriga, Maria Michela Del Viva

**Affiliations:** 1 Department of NEUROFARBA, University of Florence, Florence, Italy; 2 Department of Education, Roma Tre University, Roma, Italy; 3 Department of SARAS, Sapienza University, Roma, Italy; Federal University of Paraiba, BRAZIL

## Abstract

Educational tools in art exhibitions seem crucial to improve the cultural and aesthetic experience, particularly of non-expert visitors, thus becoming a strategic goal for museums. However, there has not been much research regarding the impact of labels on the quality of visitors’ aesthetic experience. Therefore, here we compared the impact on the cognitive and emotional experience of naïve visitors between essential and descriptive labels, through multiple objective and subjective measurements, focusing on the controversial modern art museum context. We found that, after detailed descriptions, observers spend more time inspecting artworks, their eyes wander more looking for the described elements, their skin conductance and pupil size increase, and overall, they find the content less complex and more arousing. Our findings show that people do receive important benefits from reading detailed information about artworks. This suggests that elaborating effective labels should be a primary goal for museums interested in attracting a non-expert public.

## Introduction

Over the last few years, museums have seen a significant increase in specific attention to the quality of visitors’ experience [1]. Understanding the behavior of the public, their needs, expectations, and learning processes, is now a prerequisite for the development of any project addressing the enhancement and communication of heritage. In this context, the pandemic crisis has made even more evident the need to pursue research and experimentation initiatives aimed at identifying tools and conditions useful for improving the quality of the cultural and aesthetic experience. The beneficial and soothing effect of contact with artworks has been recognized by the World Health Organisation (WHO) and recently reaffirmed by the Organisation for Economic Co-operation and Development itself [2], as an important factor in the prevention of diseases and in increasing the state of well-being of the population.

For these reasons, museums should focus their attention not only on “what” is exhibited but also on “how” works are exhibited and explained and try to adopt policies for reaching the large public of non-expert visitors. In this framework, basing strategic choices only on qualitative data rather than scientific evidence may not ensure reliable results.

In the last few years, studies have focused specifically on the quality of the visitor experience in terms of psychological and cognitive satisfaction [3]. Most studies on empirical aesthetics have been conducted in laboratories, assessing the experience with questionnaires [4, 5]. For example, Nadal and colleagues (2010) explored the influence of complexity, degree of abstraction, and artistry on beauty appreciation of artistic stimuli using multiple subjective rating scales [5]. Other studies also measured psychophysiological parameters in response to pieces of art, such as skin conductance, heart rate, eye movements, and pupillary response [6–9]. These parameters are known to reflect emotional and cognitive processes and they could be considered measures of individual reactions to artworks. For instance, skin conductance is a sensitive marker of individual meaningful events related to emotion, novelty, or attention, therefore it can be considered a “particularly pertinent window on the mind, when subjectively reported experience is not possible” [10]. In laboratory settings, it has also been found that gazing behavior and pupillary responses reflect the internal state of the observer in terms of attention, pleasure, understanding, familiarity, imagination, cognitive effort, and subjective interpretation of complex visual stimuli [6–8, 11].

Some laboratory studies, focused on the effect of artworks’ title and labels on the aesthetic experience, found that elaborative titles congruent with the content of the paintings, as well as descriptive information, facilitate the comprehension of the artworks and increase aesthetic appreciation [12–18].

Although the importance of these studies is largely recognized, recent research on art perception showed that when moving from the lab to the museum, looking at art becomes far more engaging and satisfying [19–23]. For example, original artworks in museums were liked more, viewed longer, and found more arousing compared to their digital reproductions in the laboratory [19, 24]. Also, according to Mastandrea and colleagues (2009), one aspect that characterizes visitor experience and expectation for museums of ancient and modern art was to see the work in person [25]. Therefore, research should be conducted in the real context where art is exhibited because the originality of the artworks together with the exhibition display contribute to the complexity of the aesthetic experience.

For these reasons, more recently, some studies have been conducted inside museums, mainly through observing visitors’ behavior in free-choice setting conditions and administering questionnaires after the visit, but also recording psychophysiological parameters thanks to advanced psycho-physiological portable devices [26–30]. Recent studies have also analyzed visitors’ pathways and experiences in relation to the arrangement of the exhibition [26, 31, 32]. For example, Reitstätter and colleagues (2020) investigate how the rearrangement of a museum influences the way people see and experience art, combining mobile eye tracking, subjective mapping, and a questionnaire [26]. In particular, regarding the introduction of interpretive labels in the museum setting, they wonder how visitors combine looking at art and reading labels, finding that the introduction of new labels provides benefits to artworks’ viewing time and that visitors’ engagement with the artworks was deeper, as assessed by post-visit exhibition verbal reflections [26, 33]. A more recent study investigated the role of the presence and consistency of titles influences visual exploration of artworks, finding that consistent titles produce longer saccade durations and amplitudes than untitled artworks [34].

Although the evidence suggests that the educational tools in museums may be crucial to improve the process of understanding, appreciation, and promoting individual well-being [26, 35], their role has been challenged and some museums have chosen to reduce or even eliminate explanations and labels [36] in the attempt to make the experience more emotional and less cultural-driven [37]. Scientifically evaluating the impact of labels on the perception and understanding of artworks can thus contribute to enhancing the engagement of museums in developing the quality of visitors’ experience and the efficacy of their educational offer [26].

This is particularly relevant for modern/contemporary art museums and visitors with poor art training. Non-expert people usually prefer figurative paintings compared to abstract ones [18, 25, 38–40], since their content is very often ambiguous and indefinite, compared to figurative art, where the objects represented are clearly recognizable. Indeed, appreciation is correlated to the understanding of artworks [15], and incomplete comprehension may lead to visitors’ disappointment and potentially discourage further museum visits [13].

The exploratory studies described above have delivered remarkable results and present the advantage of large sample sizes due to working with regular museum visitors. However, they do not allow measuring, with accuracy and reproducibility, the very specific cognitive and emotional processes that occur in the observer while looking at artworks as a function of specific variables, such as the labels provided by museums. Also, none of the studies investigating the impact of different labels have recorded multiple physiological and behavioral parameters in the context of a real art exhibition. Therefore, here we aim to conduct a comprehensive study to test whether descriptive labels improve the aesthetic experience, by combining multiple objective and subjective measurements in a structured experimental protocol in the very context of a modern art museum.

To this purpose, we specifically tested the impact of essential and more descriptive written labels [41] on the fruition of XX-XXI century paintings, for which the lay public expresses a lot of difficulty and perplexity in understanding and appreciating the content. We measured psychophysiological (skin conductance, heart rate, pupillary response, eye movements) and behavioral (viewing time, questionnaires) parameters in a group of art-naïve participants while looking at the artworks with different types of labels. Participants assigned to the experimental condition experienced the artworks with essential labels during a first visit and with descriptive labels during a second visit (intra-subject design). To control that the effects can be actually attributed to descriptive labels and not to the double exposure to paintings and essential labels, which could lead to familiarity effects, we introduced a control condition, in which essential labels were shown to an additional sample of participants during both sessions. We hypothesize that descriptive labels can influence both aesthetic emotional reactions and cognitive judgments [42]. Indeed, we expect increased skin conductance, heart rate, and pupillary dilation, due to changes in physiological arousal and emotional response [30, 43–48]. Furthermore, we expect that descriptive labels yield a more detailed visual inspection and prolonged viewing of paintings, leading to a better understanding of artworks revealed by higher questionnaire scores.

The outcome of this study could be of interest to museum operators, which can receive useful insight to offer more educational, descriptive, and interesting visits to a wider public.

## Materials and methods

### Participants

Thirty healthy volunteers participated in the present study (aged 21–30 years, M = 23.60, SD = 0.44); randomly assigned to the experimental (twenty observers) or the control condition (ten observers). Prior to the experiment, we collected information about participants’ personal data, art historical background, and art expertise. All selected participants had normal or corrected-to-normal visual acuity, did not take any type of medication, did not present any brain damage, and were free of cognitive disorders. All participants were university students (not art students) naive to the purpose of the experiment, with high-school level art history background. None of them were painters. On average, they had visited museums or art exhibitions only 1 or 2 times in the last year and they did not read art-related blogs, magazines, or books. To measure participants’ artistic preferences for different art types, items (e.g., “how much do you like abstract art?”) were rated with a 5-point Likert scale. On average, they like “figurative” art significantly more than “abstract” art (mean score = 3.5 ± 0.2 vs. 2.7 ± 0.1; *t*(29) = 1.8, *p*<0.05). Participants were mostly unfamiliar with the paintings and their authors: they only knew the author Mirò (14 over 30), and only two of them were familiar with the painting used. All participants were covid-free.

### Ethics

Experimental procedures were approved by the local ethics committee (Comitato Etico Pediatrico Regionale–Azienda Ospedaliero-Universitaria Meyer–Firenze FI) and are in line with the Declaration of Helsinki. Written informed consent was obtained from each participant prior to their inclusion in the study.

### Setup

Pupil and gaze data were recorded by means of a wearable eye tracking headset (Pupil Core from Pupil Labs, Berlin, Germany), composed of two eye cameras (200Hz) and a world camera (60Hz). The device was USB-connected to a MacBook Pro running a dedicated software (Pupil Capture, version 3.5.7) that enabled real-time data capture, camera recording, and calibration routines for natural conditions. A wearable wireless device equipped with high-quality data sensors (E4 wristband from Empatica Inc, Boston, USA) was used to acquire electrodermal activity (EDA, 4Hz) and heart rate (HR, computed in spans of 10 seconds) measures. The internal memory of E4 allowed us to record data continuously during the daily session (about 30 minutes per participant). The E4 device also included the possibility to press a central button during the session to mark the times of our events of interest (“tags”).

### Stimuli

The present study was conducted in the “Roberto Casamonti Collection” (https://collezionerobertocasamonti.com), a modern and contemporary art (XX-XXI century) private museum, hosted in Palazzo Bartolini Salimbeni in Florence. During each session, participants were required to follow the visit path indicated by the experimenter and stop in front of eight selected paintings. The presentation order was the same for all participants, following the position of the artwork in the exhibition.

Experimental paintings were selected before data collection, excluding those representing human figures, those totally black or white, and those that were too small or too big to be framed by the Pupil Core world camera. For each selected painting, we set an adequate distance at which observers had to stop for observation, such that each one subtended a visual angle of about 21°x15°. The paintings’ physical luminance was measured at five different points of the canvas (top-left and top-right, lower-left and lower-right, and in the center) that were averaged in a single value. Five paintings resulted in the range between 11–31 cd/m2 (16 cd/m^2^ on average), two were darker (5 and 10 cd/m^2^, 7.5 cd/m^2^ on average) and one lighter (116 cd/m^2^). We then created three luminance control stimuli (30x42cm uniform-colour canvas) for the three different levels of luminance, in order to measure baseline individual pupil diameter to those luminances.

To see all selected paintings, see **S1 Table**.

### Conditions

Participants were divided into two groups: twenty of them participated in the experimental condition and ten participated in the control condition. Both conditions consisted of two sessions at the museum on two different days, at least one month apart (on average, the second session was carried out five/six weeks after the first). In the first session, all participants were presented with essential labels (i.e., author, title, year, and technique) before seeing the paintings. In the second session, experimental participants were provided with descriptive labels (i.e., author, title, year, technique, and description of the painting’s content and the technique), whereas control participants were shown the same essential label as in the first session. See **S1 Table** for all essential and descriptive labels.

### Procedure

At the beginning of each session, participants wore the instruments and familiarized with them in a dedicated room. Before starting the visit, they were positioned in front of the three luminance-control stimuli and asked to look at each for ten seconds. They were then instructed to follow the experimenter from one painting to the next and to press the timestamp button on the Empatica wristband (“tag”) every time they started and stopped reading a label and looked at the painting. A two-second red light was displayed on the wristband after each button press; therefore, the experimenter could check that participants correctly pressed the tag when needed (and promptly reminded him/her to press it in case of occasional forgetfulness).

**Fig 1:**
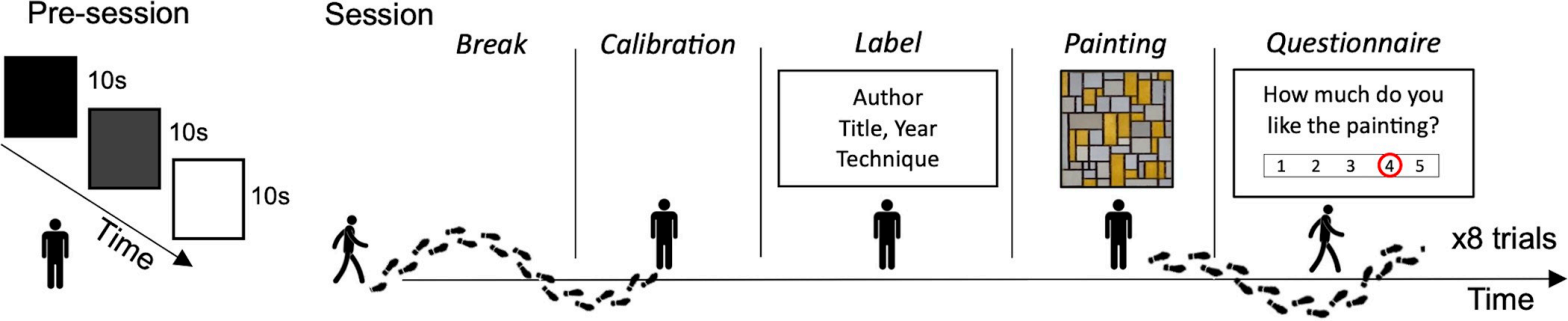
Experimental procedure. Vertical lines in the figure represent the “tags”, which are the moments that delimit when participants start and stop the different phases of a trial. The painting in the figure is copyright-free; it has been shown only for illustrative purposes and it is not part of the set of stimuli used here. Copyright laws prohibit the reproduction of the paintings used as stimuli in this experiment, but they can be viewed at the links reported in S1 Table.

After the pre-session measurements, observers reached the first painting indicated by the experimenter and stood in front at the preset distance. Once the eye tracker was calibrated (we used an 8-points *natural-features* calibration routine), participants could read the label. The label, written on a sheet of paper, was shown by one experimenter standing in front of the participant. Then participants looked at the painting for as long as they wanted, pressing a “tag” when they started and stopped reading and observing. After they finished observing the painting, the experimenter asked them some questions about the artwork and reported the answers on a notepad. The questionnaire required the participants to score on a 5-points Likert scale the following items: complexity, comprehensibility, title informativeness, positive emotions, negative emotions, appreciation, interest, and curiosity for other works of the same author. Participants were also asked to report if the paintings and the authors were familiar or unfamiliar. Then participants continued the visit to the next selected painting. For a schematic representation of the experimental procedure see **Fig 1**.

### Data processing and statistical analysis

Physiological parameters were recorded from the start to the end of each museum session, so that, for each participant, we obtained a continuous recording of about 25–30 minutes per session. Raw data from the wristband and the eye tracker were extracted in.csv format and synchronized through an ad-hoc procedure in Matlab (R2020b version; Natick, Massachusetts: The MathWorks Inc.). The timestamps (“tags”) were converted to real times and used to delimitate our events of interest.

The participants’ artistic preferences for different art types, rated with a 5-point Likert scale, were calculated. We performed paired sample *t*-tests across subjects to assess significant differences between art types.

The reading time of each label was calculated as the difference in seconds between the two tags indicating the start and the end of the observer’s reading. The time of viewing of each painting was calculated as the difference in seconds between the two tags indicating the start and the end of the observer’s visualization. Thus, the artworks’ viewing time does not include the time spent reading the labels. For each participant, the viewing times of all the paintings were averaged together. Then, the times of all participants were averaged. To compare the average viewing time between the essential and the descriptive label sessions, and between the two control sessions with essential labels, a two-way ANOVA analysis with within-subjects factor *session* (two levels: first vs. second session) and between-subjects factor *condition* (two levels: experimental vs. control condition) was done. *P*-values obtained from post hoc analyses were adjusted using the Bonferroni correction. Effect sizes of the differences were estimated by eta-squared statistics (*η^2^*) with 95% confidence intervals. The average viewing time of each participant was also correlated (Pearson linear-correlation coefficient) to the information collected prior to the experiment about their art expertise and artistic preferences.

For each questionnaire item, administered during the experiment after viewing each painting, the scores assigned by each participant to each painting were averaged together. To compare average scores between the essential and the descriptive label conditions, and between the two control sessions with essential labels, paired-sample Wilcoxon signed-rank tests across paintings were performed. The effect size of differences between conditions was estimated by Rank-Biserial correlation (r_rb_) with 95% confidence intervals. Also, individual scores for each painting were related to the corresponding EDA, HR, and pupil responses to calculate the Pearson linear-correlation coefficient.

For measuring the changes in EDA and HR responses induced by the painting, we normalized each trace considering as baseline the average EDA/HR value in the last three seconds before looking at the artwork. Pupil diameter was converted from pixel to millimeters by measuring the eye tracker recording of a 4 mm artificial pupil, positioned at the location of the observer’s eyes. For measuring pupil size variations induced by paintings, each trace was normalized, considering as baseline the average pupil diameter in response to the luminance-matched control stimulus presented before each session (**Fig** 1). To produce plots as a function of time, for each painting, normalized traces were averaged for each recorded time across participants. Since viewing time changes across participants, only means including at least five participants were considered. This process has led to average recordings where the initial values include all participants, whereas the last values include only participants with long viewing times. Finally, average traces for each painting were averaged together.

To perform statistical analysis, for each normalized trace of each participant for each painting, the average value and the root mean square error (RMSE) during the whole viewing time were calculated. The means and RMSE of all parameters were compared with two-way ANOVA analyses with within-subjects factor *session* (two levels: first vs. second session) and between-subjects factor *condition* (two levels: experimental vs. control condition). *P*-values obtained from post hoc analyses were adjusted using the Bonferroni correction. Effect sizes of the differences were estimated by eta squared statistics (*η^2^*) with 95% confidence intervals.

Since the gaze is recorded through a head-centred camera, and thus subjected to head movements, to analyze the gaze pattern we adopted a manual procedure [26, 27]. We subdivided each painting into 25 equally sized areas so that each area subtended a visual angle of about 4°x3° in each painting. All video recordings were extracted by using the Pupil Player software and each position of the gaze shown in the videos was manually converted to a position in one of the 25 areas. Then we counted how many times each area had been watched by each participant. To compare the difference of fixations in the descriptive vs. essential label or between the two control sessions for each of the 25 areas a heat map for each painting was calculated as follows. Since the number of fixations in the two sessions is different, the proportion of fixations in each area (with respect to the total number of fixations in that condition) in the different sessions was calculated and then their difference was computed. For representational purposes, the distribution of differences between the second and first session was binned into five density levels: one (the middle) corresponding to the median of the distribution (equal density of fixations), the others corresponding to quartiles of the distribution.

To study the distribution of fixations as a function of eccentricity, three eccentricities were considered for the whole canvas area: central area 0°-7°, nearby periphery 7°-14°, and periphery 14°-21°. Then the number of fixations for each eccentricity and for all observers was calculated. Finally, for each eccentricity, the difference between fixations in the descriptive label and essential label sessions and between the two control sessions was calculated. Comparisons of these values between different eccentricities were done with paired-sample two-tailed *t*-tests. The effect size of differences between conditions was estimated by Cohen’s *d* statistics with 95% confidence intervals.

## Results

**Fig 2:**
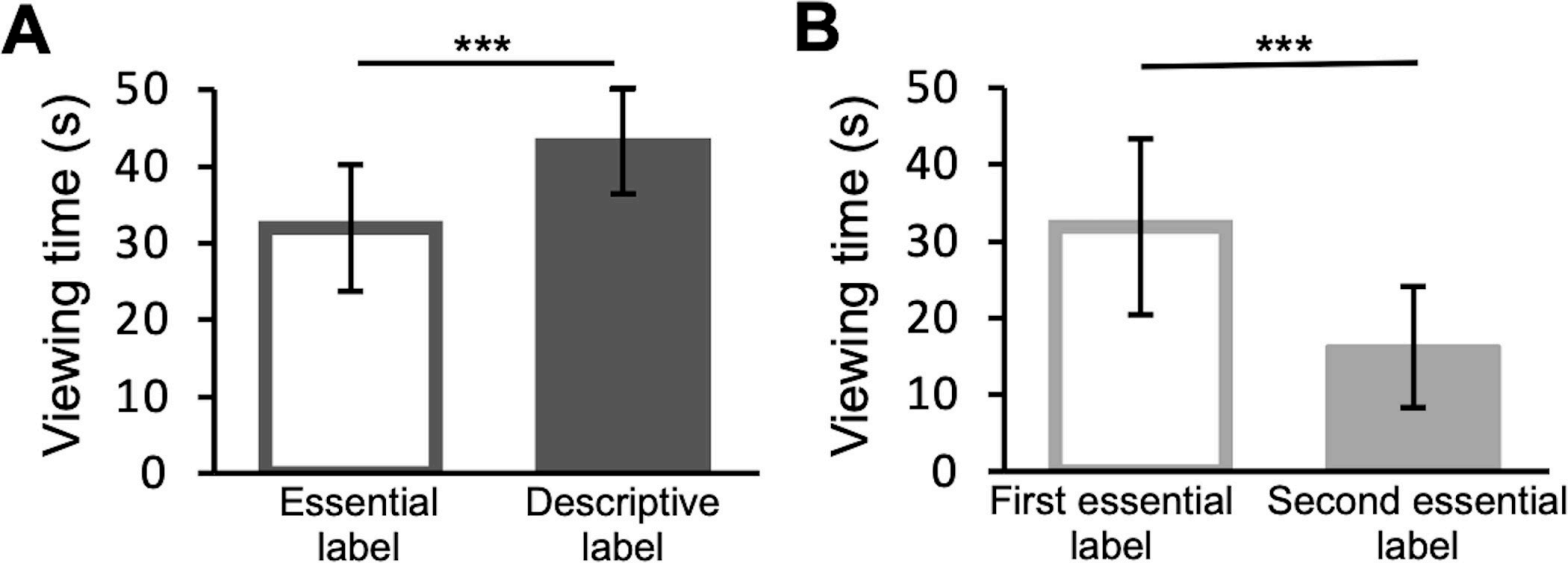
Viewing time. (A) Experimental condition (B) Control condition. The bars show the viewing time of the paintings averaged across participants. Intervals. Errors are 95% confidence intervals. Significance levels refer to post hoc comparisons: ***p<0.001.

Considering average time viewing, ANOVA analysis reveals no significant difference between sessions (F(1,24) = 1.04, *p*>0.05, *η*^*2*^ = 0.003). On the other hand, there is a significant effect of conditions (F(1,28) = 5.2, *p*<0.01, *η*^*2*^ = 0.1) and of the interaction between factors (F(1,28) = 40.9, *p*<0.001, *η*^*2*^ = 0.1). Indeed, in the experimental condition, the average time spent viewing the paintings is significantly lower in the first than in the second session, thus observers’ viewing time is significantly longer after reading a descriptive label compared to an essential label (post-hoc comparisons; t = -4.6, *p*<0.001; **Fig 2A)**. On the contrary, in the control condition, the average viewing time is significantly longer in the first session than in the second session with essential labels (post-hoc comparisons; t = 4.5, *p*<0.001; **Fig 2B)**. Viewing times in the first sessions of the experimental and control conditions are comparable *s*ince both groups read essential labels during the first visit (post-hoc comparisons; t = -0.013, *p*>0.05). Average viewing times of each painting in the experimental and control condition are shown in **S1 Fig**. Average reading times of labels of each painting in the experimental and control condition are reported in **S2 Table.**

A positive correlation emerges between participants’ preference for abstract art (rated with a 5-point Likert scale before the experiment; see Methods section) and the average viewing time of paintings during the first visit (Pearson linear-correlation; r(28) = 0.56, *p*<0.01).

**Fig 3:**
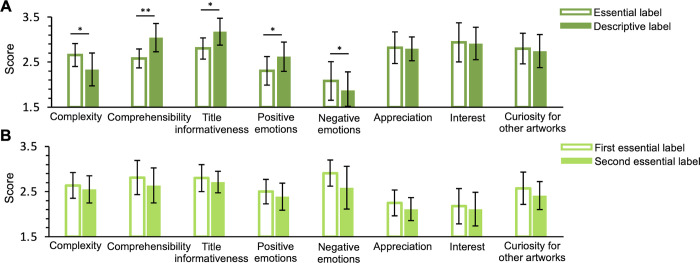
Questionnaire scoring (A) Experimental condition (B) Control condition. The bars show the average score that participants attributed to the different items of the questionnaire mediated across paintings. Errors are 95% confidence intervals. Significance levels refer to paired-sample Wilcoxon signed-rank test: *p<0.05; **p<0.01.

For the experimental condition, questionnaire’ scoring reveals that descriptive labels, compared to essential labels, influence with very strong effect sizes several dimensions (**Fig 3A)**: perception of artwork’s complexity decreases (Paired-sample Wilcoxon signed-rank test; W(7) = 36.01, *p*<0.05, r_rb_ = 1.00, 95% CI [1.00, 1.00]), contents’ comprehensibility increases (W(7) = 0.01, *p*<0.01, r_rb_ = 1.00, CI [-1.00, -1.00]), the title looks more informative (W(7) = 2.01, *p*<0.05, r_rb_ = 0.89, CI [-0.97, -0.56]), positive emotions increase (W(7) = 0.01, *p*<0.05, r_rb_ = 1.00), while negative emotions decrease (W(7) = 35.01, *p*<0.05, r_rb_ = 0.94, CI [0.76, 0.99]). Aesthetic appreciation, interest, and curiosity for other artworks of the same authors do not change significantly between different labels. No significant questionnaire differences were found between sessions in the control condition (**Fig 3B**).

**Fig 4:**
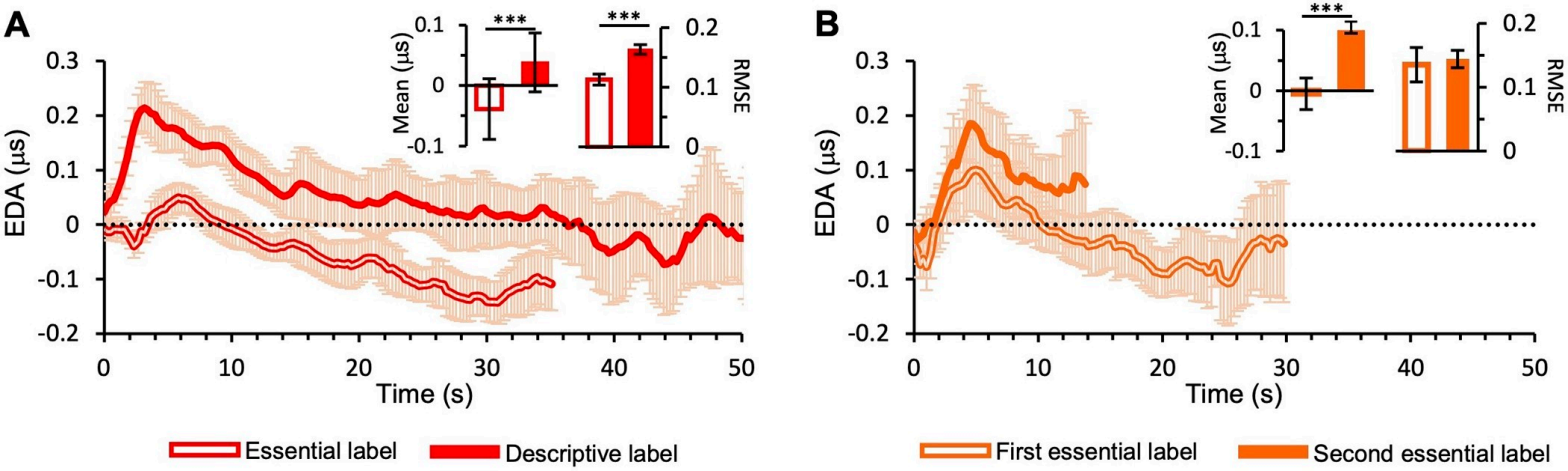
Electrodermal activity. (A) Experimental condition (B) Control condition. The lines show skin conductance recording over time, averaged across paintings and participants. In each panel, the bar graph on the left shows the mean EDA response averaged across paintings; the bar graph on the right shows RMSE averaged across paintings. Errors are 95% confidence intervals. Significance levels refer to post-hoc comparisons: **p<0.01, ***p<0.001.

Regarding the average EDA response, ANOVA analysis reveals a significant difference between conditions (F(1,14) = 12.3, *p*<0.01, *η*^*2*^ = 0.2) and sessions (F(1,14) = 27.7, *p*<0.001, *η*^*2*^ = 0.3). Indeed, EDA in the second session increases during the first seconds of painting viewing more than with essential labels and remains higher during the whole viewing time (line graph in **Fig 4A**), as well as in the control condition (see line graph in **Fig 4B).** The average EDA response is significantly higher both for experimental (see bar graph in **Fig 4A—left panel)** and control condition (see bar graph in **Fig 4B—left panel).** No significant interactions emerge between factors (F(1,14) = 0.3, *p*>0.05, *η*^*2*^ = 0.004). For RMSE, ANOVA shows a significant effect of the session (F(1,14) = 26.7, *p*<0.001, *η*^*2*^ = 0.3), no effect of the condition (F(1,14) = 0.07, *p*>0.05, *η*^*2*^ = 0.002), and a significant interaction between the two factors (F(1,14) = 10.9, *p*<0.01, *η*^*2*^ = 0. 1). Particularly, post-hoc comparisons reveal a statistical difference between the first and the second session during the experimental condition (t = -6.1, *p*<0.001; **Fig 4A—right panel**). However, no statistical differences in the average RMSE are found in the control condition (see **Fig 4B–right panel**). Average EDA responses to each painting in the experimental and control condition are shown in **S2 Fig**.

Neither session (F(1,14) = 0.0, *p*>0.05, *η*^*2*^ = 0.0), condition (F(1,14) = 0.07, *p*>0.05, *η*^*2*^ = 0.002) or their interaction (F(1,14) = 1.3, *p*>0.05, *η*^*2*^ = 0.05) affect heart rate response.

**Fig 5:**
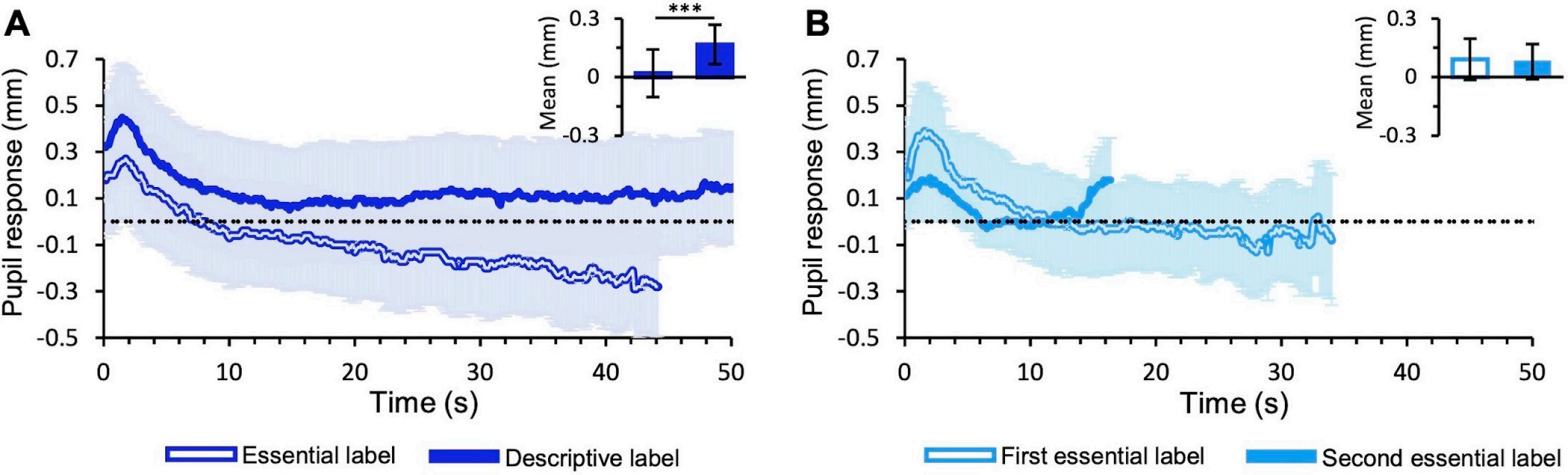
Pupillary response. (A) Experimental condition (B) Control condition. The lines show pupil response over time averaged across paintings and participants. The bar graph shows the mean pupil response averaged across paintings. Errors are 95% confidence intervals. Significance levels refer to post-hoc comparisons: ***p<0.001.

Considering pupillary responses, ANOVA reveals no statistical differences between conditions (F(1,14) = 0.003, *p*>0.05, *η*^*2*^ = 0.0). On the contrary, there is a significant effect of the session (F(1,14) = 5.9, *p*<0.05, *η*^*2*^ = 0.02) and of the interaction between factors (F(1,14) = 8.4, *p*<0.05, *η*^*2*^ = 0.02). Indeed, in the experimental condition, pupillary responses differ between the two sessions: the pupil is always more dilated with descriptive than with essential labels (line graph in **Fig 5A**). Average pupil variation is positive and statistically higher with descriptive labels than with essential labels (post-hoc comparisons; t = -6.1, *p*<0.001; bar graph in Fig 5A), but no differences are found in the control condition (**Fig 5B**). Average pupillary responses to each painting in the experimental and control condition are shown in **S3 Fig**.

There are no correlations (Pearson linear-correlation) between individual psychophysiological responses to paintings and corresponding questionnaire scores (all *p*>0.05). Also, the responses are not affected by familiarity with the paintings (i.e., the responses to the most familiar painting—*Femme*, Miró, 1977–1978 –are the same as those found for all the other unknown stimuli).

**Fig 6:**
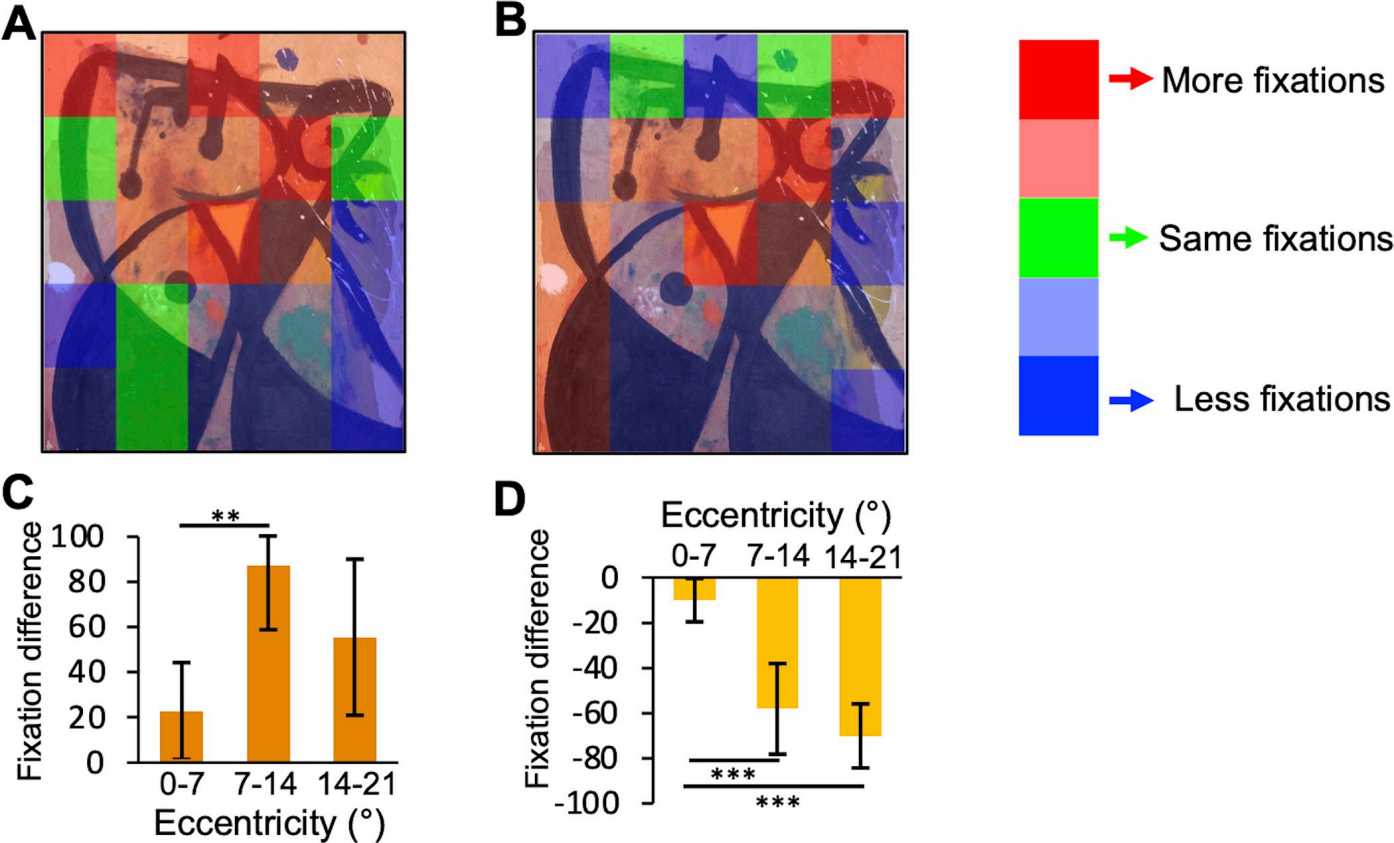
Gaze patterns and fixations. **(A) Example of gaze pattern in a painting: Essential label vs descriptive label.** The heat map shows the different proportion of fixations in the two label conditions for the painting *Femme* (Miró, 1977–1978), that can be viewed at the link reported in S1 Table; courtesy of Successiò Mirò (2023). Red: higher proportion of fixations with descriptive labels; green: same proportion of fixations; blue: lower proportion of fixations with descriptive labels. **(B) Example of gaze pattern in a painting: First session vs second session both with essential label.** The heat map shows the different proportion of fixations in the two label conditions for the painting *Femme* (Miró, 1977–1978); courtesy of Successiò Mirò (2023). Red: higher proportion of fixations in the second session; green: same proportion of fixations; blue: lower proportion of fixations in the second session. **(C) Spatial distribution of fixations in the experimental condition.** The bar graph shows the difference in the number of fixations between descriptive and essential label conditions at three eccentricities. Errors are 95% confidence intervals. Significance levels refer to paired-sample *t*-test. **(D) Spatial distribution of fixations in the control condition.** The bar graph shows the difference in the number of fixations between the second and the first session at three eccentricities. Errors are 95% confidence. Significance levels refer to paired-sample *t*-test: **p<0.01, ***p<0.001. See Data Processing section for data analysis details.

Eye movements analysis during painting viewing highlights some differences across label conditions (see **Fig 6**). First, gaze patterns result to be related to the painting’s description. For example, eye movements in the Miró painting (*Femme;* 1977–1978) are directed toward the elements depicting feminine body parts, as described in the descriptive label of the experimental condition (see **S1 Table** and the heatmap in **Fig 6A**). Instead, in the control condition without a description, the eyes are less directed towards salient paintings’ elements (see the heat map in **Fig 6B**). Since participants in the experimental condition spent more time looking at the painting with descriptive labels, the number of fixations in this condition is higher (275±30 vs 220±37, on average). On average, after reading the description, participants tend to fixate more in the closer periphery (7°-14°) than the painting’s centre (0°-7°; Paired sample *t*-test; t(7) = -4.05, *p* < .01, *d* = -1.43, 95% CI [-2.42, -0.41]; see the bar graph in **Fig 6C**). On the other hand, in the control condition, the number of fixations is lower in the second session, as expected by less time spent observing the painting. Observers mainly fixate the centre of the paintings: the number of fixations in the near (7–14°) and far periphery (14–21°) is much lower than in the centre (0–7°) between the two sessions (t(7) = 5.78, *p*<0.001, *d* = 2.05, CI [0.77, 3.28] and t(7) = 5.84, *p*<0.001, *d* = 2.07, CI [0.78, 3.31] respectively; see bar graph **Fig 6D**).

## Discussion

In the present study, we compared the impact of essential and descriptive labels on the cognitive and emotional experience of naïve visitors, through multiple objective and subjective measurements, focusing on controversial modern paintings. Indeed, socio-cultural bias and stereotypes tend to twist the answers of people, often worried to be judged for their lack of expertise in art history, and this process becomes particularly relevant in the context of modern art museums, where the sense of self-efficacy and ease of visitors tend to decrease and–at the same time–the need for educational support, from the majority of the public, becomes unavoidable. People can feel a sense of frustration in front of artists and movements which they merely know and, most of all, hardly understand [13]. Our decision to work with a collection of modern art derives from these considerations and from the widely diffused commonplace that ancient art is “easier” than modern art [25, 38]. Coherently with these premises, we have decided to select a group of people who lacked any particular artistic experience and art history background: a characteristic which made our sample quite homogenous from the art-expertise point of view.

Our results show that objective and subjective responses while inspecting modern artworks change depending on the information received before experiencing the paintings. A detailed description encourages participants to spend more time observing artworks, following the information provided. It is difficult for non-experts to catch the meaning of modern artworks [49]. For example, the Miró painting may appear as a series of wide black brushstrokes with small, coloured spots. But when participants come to know that the spots outline the shape of a female body, their eyes perform a greater number of fixations on the parts depicting the figure. This suggests that the explanation provides a key to cognitive and emotive comprehension, confirmed by the subjective perception of increased positive feelings and comprehension.

The increase and variability of electrodermal activity and pupil dilation, suggest an increase in physiological arousal [10, 43]. This could be due to a deeper understanding of artworks and a higher cognitive load [50], as well as to an increase in emotional load [45]. Subjective judgments after viewing the paintings cannot further shed light on the relative contribution of the two dimensions. The increased EDA in the control condition suggests that this psychophysiological response might be modulated by the familiarity for the stimuli, which has been linked to faster processing and higher preference for familiar stimuli compared to novel ones [51–53]. However, the phasic EDA activity changes when subjects have read the painting description, maybe related to focusing on different elements, while it does not change in the control condition [44].

Our results also show that participants who appreciate more abstract art are the ones spending more time in front of the paintings. However, aesthetic appreciation for the specific paintings presented during the experiment does not change upon explanation. This suggests that, although labels can facilitate comprehension, this is not enough to cause an increased appreciation. We can speculate that specific art training is needed to appreciate modern artworks. Indeed, expertise in art facilitates the so-called *aesthetic fluency* [54]; a process that could lead people to better grasp the meaning of an artwork and to its aesthetic appreciation. Also, this result may be interpreted by the fact that modern/contemporary art does not have as its main objective to be "beautiful", rather to be interesting, activating, provocative, ambiguous, and meaningful.

Overall, our findings show that visitors do receive important benefits from reading detailed information about artworks. On a more general ground, art description leads to changes in aesthetic judgment and aesthetic emotion outputs, described in the Leder and colleagues’ *aesthetic experience model* [42].

Since descriptive labels are used when paintings are also seen for the second time, it could be that label-based effects may be conditional on paintings that participants are already familiar with. On the other hand, participants who visited the museum twice receiving the same essential information do not show increased satisfaction. They spent less time observing the painting and assigned the same scores to the questionnaire for understanding and appreciation of the artworks. Also, observers fixate and explore less the artworks, as expected. Except for a slight increase in electrodermal activity, variability of EDA and pupil dilation do not change in the second session. Overall, these results suggest that familiarity with the stimuli without additional information does not improve the museum experience in terms of aesthetic judgments and emotional reactions in naïve visitors, but rather causes them to pay less attention to the artworks [42].

Some of our findings can be compared to those of previous studies. The average viewing times found here with essential and descriptive labels are in line with those found in previous works using unfamiliar artworks [20, 26, 55–57]. Viewing times are generally longer for well-known paintings (e.g., “The Kiss” by Klimt; [26, 33]); however, here the only painting familiar to our participants (*Femme*, Miró, 1977–1978) received the same amount of time. Mastandrea and colleagues (2019) measured blood pressure and heart rate before and after museum visits, finding that visits to art museums decreased the level of systolic blood pressure but did not influence the heart rate [28]. This is in line with our absence of effects of labels on the HR. It has been found that the display influences the way people experience art, causing different viewing times, levels of engagement and different patterns of fixations [26, 34], as we found with different types of labels. In contrast, interests in specific artworks and art style preferences proved to be robust and independent of presentation modes. This confirms our results on aesthetic appreciation, which does not increase by introducing descriptive labels. They also found that when labels are more complex (with more text), visitors’ interpretation differs according to the information received, and so do we. Tschacher and colleagues (2012) found that artworks’ understanding was correlated with higher skin conductance variability [45]. We also found higher EDA variability with descriptive labels, even if we did not find any correlation with specific cognitive or emotional domains of the questionnaire. Increased pupil dilation, which we found after presenting a description of the artwork, has been found in some studies conducted outside the museum context, where it was associated with aspects of aesthetic emotions [11]. Note however that, none of this research has measured psychophysiological and behavioral responses during the museum visit as a function of descriptive material, that could influence the aesthetic experience.

We cannot rule out that our findings depend on the particular type of artworks involved. Further research might be undertaken in order to make comparisons between “ancient” art and “modern” art, trying to explore possible differences in reactions of visitors in front of visual languages which are perceived as more familiar or simply closer to a general “common taste”. Indeed, robust findings show that non-expert observers usually prefer figurative paintings compared to abstract or conceptual artworks [18, 25, 38–40], and that art appreciation correlates with educational level [38]. Ancient art is more easily recognizable and can result to be less anxiety-trigger, especially for non-expert visitors; nevertheless, we should not underestimate the emotional effect (in terms of involvement and gratification) that “ancient masters” can activate on the general public.

During real museum visits, visitors can go back to view some paintings they particularly like while ignoring others, they usually go back and forth between reading labels and viewing artworks, sometimes they may be in the company of other people, and they generally experience many artworks, facing the problem of museum fatigue (for a review, see [58]). In our study, we gave up these situations occurring in an optimal ecological condition in favor of reproducible and accurate measurements, with the aim of avoiding confounding variables. Also, we cannot exclude that the outcome might have been different having as participants a sample of regular museum-goers.

In the future, it would be interesting to test art-expert participants; the knowledge they already possess should be enough to understand the meaning of the artwork and have a satisfying experience without the need for informative materials. Expertise is known to lead to higher aesthetic appreciation of artworks and differences in viewing strategies, gaze patterns, fixation distributions, and even in electrophysiological correlates [44, 59–62]. Fixations should be more focused on salient parts of artworks because the meaning could be grasped even without a descriptive label. More complex is the question of aesthetic appreciation, which experts tend to underestimate in comparison to the complexity of meaning (while for naïve visitors may result as the leading value). We expect that a naïve group would receive more beneficial effects from the explanation of artworks than experts would do, as they might be influenced by their personal evaluation of the quality and amount of information received.

Overall, our work suggests that elaborating effective labels, based on scientific evidence rather than on qualitative observations, should be a primary goal for museums. Indeed, if museums aim to attract a wider public, they need to focus their attention on didactic tools provided by panels and captions, with the hope to fill the gap generated by the lack of art knowledge. This is particularly relevant for modern art which is less known and harder to understand and appreciate by non-art experts, that can otherwise perceive the museum visit as a frustrating experience due to their little art education.

## Conclusion

Aesthetic experience is a psychophysiological process which arises during artworks fruition and involving a variety of emotional and cognitive responses. Here we use multiple psychophysiological and behavioral tools to measure rigorously, in the very context of a modern art museum, the effects of explanatory texts and labels on modern art experience. Our findings show that people receive important benefits, in terms of cognitive and emotional involvement, by reading detailed descriptions of modern artworks. The outcome of our studies could be of interest to museum operators and may become instrumental for improving exhibition, website information content, and advertising material, and for achieving optimal fruition, satisfaction, and thus contribute to well-being of naive visitors as well as experts. On more general grounds our results indicate that psychophysiological changes can be an effective probe into artworks processing and interpretation, making them useful tools for the study of museum aesthetic experience.

## Supporting information

S1 Table Paintings and their essential and descriptive labels. First column: web link to the paintings used as stimuli (in the same order as presented to the visitors at the Casamonti collection). Second column: essential label read by the participants in the first experimental session and by control participants in the two control sessions (regular style: Italian version; italics style: English version). Third column: descriptive label read by the participants in the second experimental session (regular style: Italian version; italics style: English version).
(DOCX)

S2 Table Reading time of labels of each painting in the experimental and control condition. Data in the table are reading time in seconds averaged across participants.
(DOCX)

S1 Fig Viewing time of each painting. (A) Experimental condition. (B) Control condition. The bars show the viewing time of each painting (from painting 1 to painting 8, in the same order as presented to the visitors at the Casamonti collection), averaged across participants. Errors are SE across participants.
(TIF)

S2 Fig Electrodermal response to each painting. (A) Experimental condition. (B) Control condition. The bars show the EDA response to each painting (from painting 1 to painting 8, in the same order as presented to the visitors at the Casamonti collection), averaged across participants. Errors are SE across participants.
(TIF)

S3 Fig Pupillary response to each painting. (A) Experimental condition. (B) Control condition. The lines show pupil response over time to each painting (from painting 1 to painting 8, in order as presented to the visitors at the Casamonti collection), averaged across participants. Errors are SE across participants.
(TIF)
