# Supplementary material for: Psychophysiological and behavioral responses to descriptive labels in modern art museums
Source: PLoS One. 2023 May 3;18(5):e0284149. doi: 10.1371/journal.pone.0284149 (PMC10155981; doi:10.1371/journal.pone.0284149)
Supplement: S2 Table — Data in the table are reading time in seconds averaged across participants. (DOCX) [file pone.0284149.s002.docx]

| ***Paintings*** | ***Experimental condition*** | | ***Control condition*** | |
| --- | --- | --- | --- | --- |
|  | ***Essential label (s)*** | ***Descriptive label (s)*** | ***First essential label (s)*** | ***Second essential label (s)*** |
| Ebla | 13.0 | 41.9 | 15.1 | 14.9 |
| Empreinte d’un nu (f7) | 14.7 | 54.5 | 14.8 | 14.0 |
| L-1-75 | 13.2 | 45.9 | 13.9 | 13.7 |
| Empremta de cos | 13.6 | 46.2 | 14.8 | 14.4 |
| Femme | 12.3 | 43.5 | 13.5 | 12.6 |
| Still life | 12.1 | 43.9 | 13.8 | 12.5 |
| Canto dal mare | 12.6 | 45.8 | 15.4 | 13.1 |
| Cuore pompeiano | 11.8 | 42.4 | 14.5 | 12.3 |
