# Supplementary figures and images for: Psychophysiological and behavioral responses to descriptive labels in modern art museums
Source: PLoS One. 2023 May 3;18(5):e0284149. doi: 10.1371/journal.pone.0284149 (PMC10155981; doi:10.1371/journal.pone.0284149)

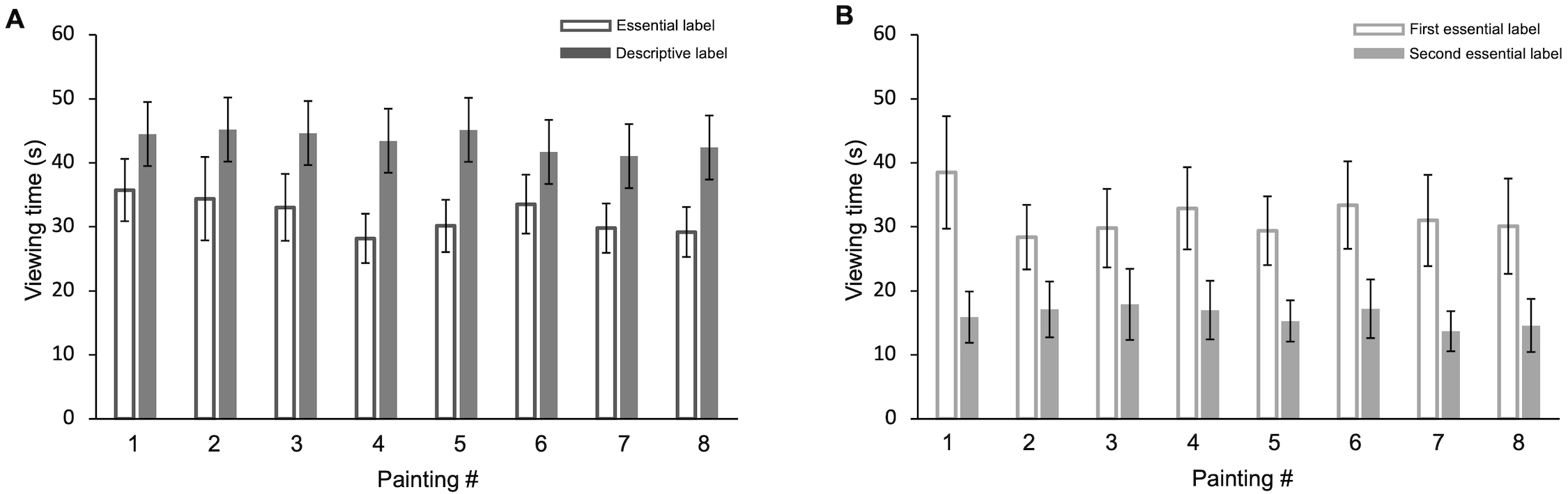

Supplement: S1 Fig — (A) Experimental condition. (B) Control condition. The bars show the viewing time of each painting (from painting 1 to painting 8, in the same order as presented to the visitors at the Casamonti collection), averaged across participants. Errors are SE across participants. (TIF) [file pone.0284149.s003.tif]

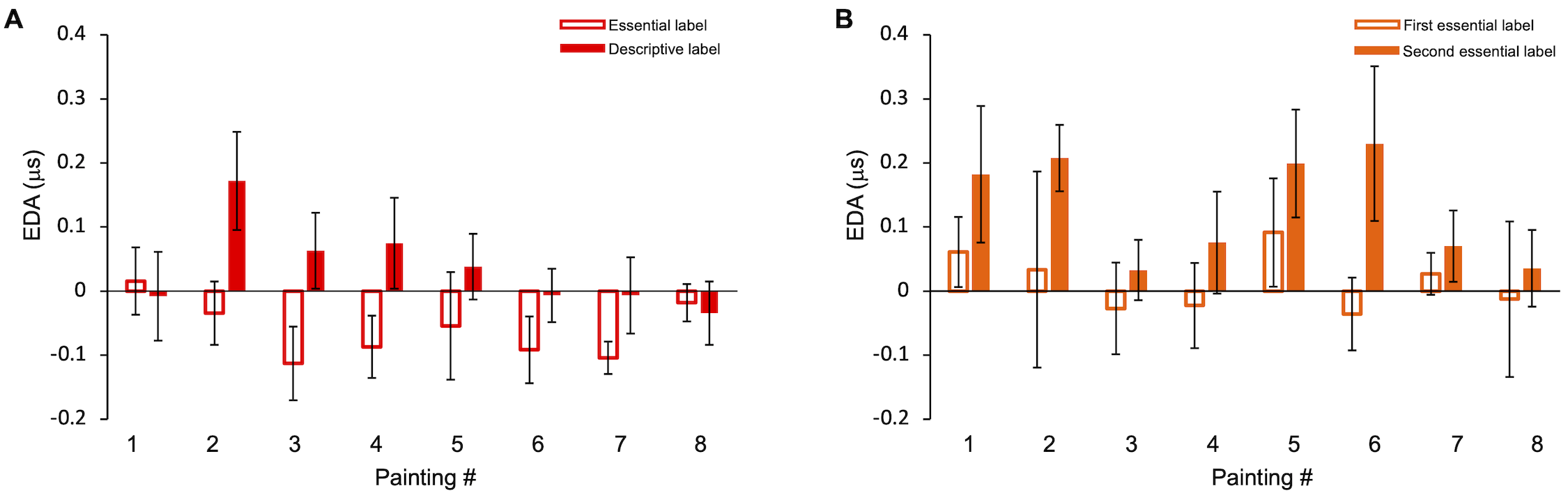

Supplement: S2 Fig — (A) Experimental condition. (B) Control condition. The bars show the EDA response to each painting (from painting 1 to painting 8, in the same order as presented to the visitors at the Casamonti collection), averaged across participants. Errors are SE across participants. (TIF) [file pone.0284149.s004.tif]

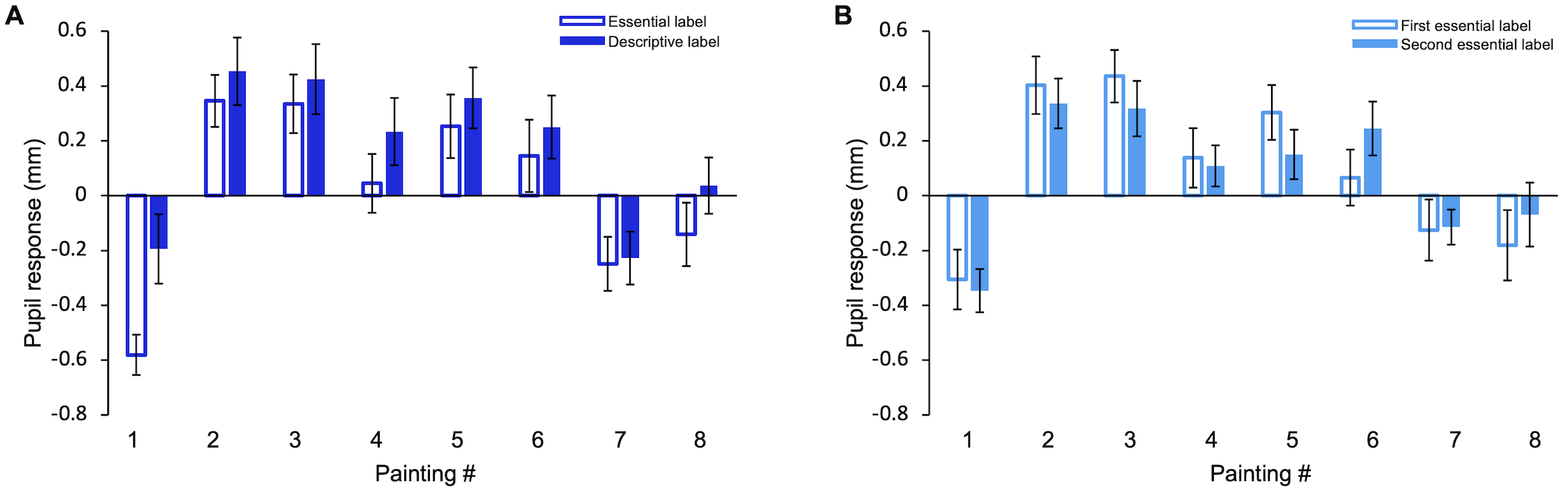

Supplement: S3 Fig — (A) Experimental condition. (B) Control condition. The lines show pupil response over time to each painting (from painting 1 to painting 8, in order as presented to the visitors at the Casamonti collection), averaged across participants. Errors are SE across participants. (TIF) [file pone.0284149.s005.tif]
